# Exploring the Superior Anchoring Performance of the Two-Dimensional Nanosheets B_2_C_4_P_2_ and B_3_C_2_P_3_ for Lithium–Sulfur Batteries

**DOI:** 10.1021/acsomega.2c03898

**Published:** 2022-10-20

**Authors:** Hiba Al-Jayyousi, Mathan Kumar Eswaran, Avijeet Ray, Muhammad Sajjad, J. Andreas Larsson, Nirpendra Singh

**Affiliations:** † Department of Mechanical Engineering, Khalifa University of Science and Technology, Abu Dhabi 127788, United Arab Emirates; ‡ SRM Research Institute, SRM Institute of Science and Technology, Kattankulathur 603203, Tamil Nadu, India; § Department of Physics, Indian Institute of Technology Roorkee, Roorkee 247667, India; ∥ Department of Physics, Khalifa University of Science and Technology, Abu Dhabi 127788, United Arab Emirates; ⊥ Applied Physics, Division of Materials Science, Department of Engineering Sciences and Mathematics, Luleå University of Technology, Luleå SE-97187, Sweden; # Center for Catalysis and Separations, Khalifa University of Science and Technology, Abu Dhabi 127788, United Arab Emirates

## Abstract

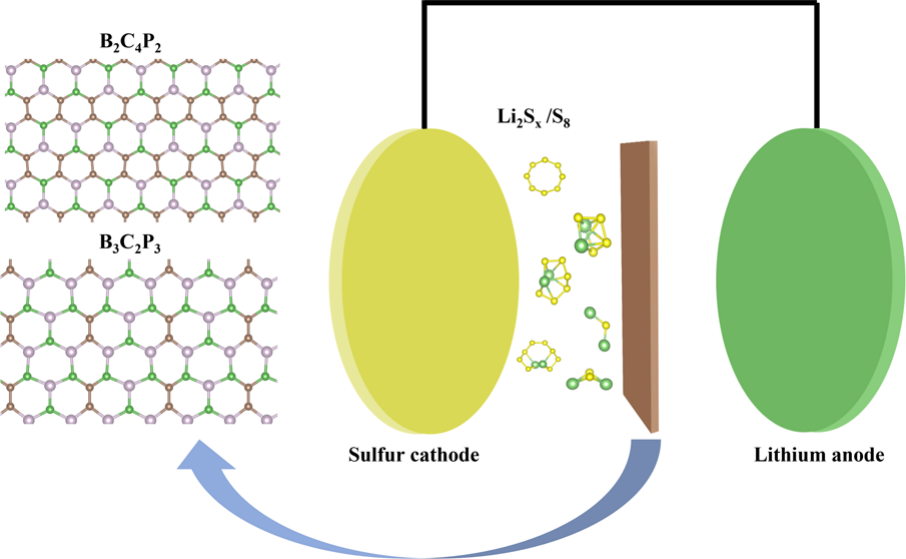

Potential anchoring materials in lithium–sulfur batteries help overcome the shuttle effect and achieve long-term cycling stability and high-rate efficiency. The present study investigates the two-dimensional nanosheets B_2_C_4_P_2_ and B_3_C_2_P_3_ by employing density functional theory calculations for their promise as anchoring materials. The nanosheets B_2_C_4_P_2_ and B_3_C_2_P_3_ bind polysulfides with adsorption energies in the range from −2.22 to −0.75 and −2.43 to −0.74 eV, respectively. A significant charge transfer occurs from the polysulfides, varying from −0.74 to −0.02*e* and −0.55 to −0.02*e* for B_2_C_4_P_2_ and B_3_C_2_P_3_, respectively. Upon anchoring the polysulfides, the band gap of B_3_C_2_P_3_ reduces, leading to enhanced electrical conductivity of the sulfur cathode. Finally, the calculated barrier energies of B_2_C_4_P_2_ and B_3_C_2_P_3_ for Li_2_S indicate fast diffusion of Li when recharged. These enthralling characteristics propose that the nanosheets B_2_C_4_P_2_ and B_3_C_2_P_3_ could reduce the shuttle effect in Li–S batteries and significantly improve their cycle performance, suggesting their promise as anchoring materials.

## Introduction

Due to high efficiency, high capacity, and environment-friendly characteristics, lithium–sulfur (Li–S) batteries continue to develop as the most promising rechargeable energy source for the near future.^1,2^ Compared to commercial lithium-ion batteries, Li–S batteries show a higher theoretical capacity of 1675 mAh g^–1^ and specific energy of 2600 Wh kg^–1^.^3−6^ In addition to the higher energy content, a lower cost is associated with Li–S batteries since sulfur cathodes are cheap and have many merits including abundant resources.^7,8^ However, several challenges that limit the commercial implementation of Li–S batteries need to be addressed. These limitations include low active material utilization, high volume changes in the charge/discharge process, and formation of lithium dendrites, which may lead to short circuits during cycling, termed as the shuttle effect.^2,9,10^ This notorious and intractable effect is due to the migration of sulfur intermediates between the positive and negative electrodes in the charging and discharging process.^11,12^ In this process, lithium polysulfides (LiPSs) Li_2_S*_x_*, 2 < *x* ≤ 8, are formed because sulfur atoms react with the transferred lithium ions that risk transportation back to the Li electrode upon recharging, which leads to depletion of S in the S electrode and low Coulombic efficiency along with fast capacity fading.^13,14^ Over the past few years, considerable efforts to suppress the shuttle issue have been reported, including electrolyte tuning,^15,16^ cathode functionalization,^17−19^ and introducing anchoring materials that block the diffusion pathway of the polysulfides.^20−24^ As a result, promising anchoring materials, which can activate strong interactions with the Li_2_S*_n_* species, have been noticed as an adequate way to overcome these problems and achieve long-term cycling stability and high-rate performance. The anchoring materials are classified as weak (chlorides), moderate (sulfides), and strong (oxides) anchoring materials. Therefore, the entrapment effect is related to the chemical architecture of the anchoring materials. Ideal anchoring materials should have the following criteria: (a) display moderate binding energies for the Li_2_S*_n_* species, (b) have sufficiently active regions, and (c) be lightweight; these would lead to sufficiently confined polysulfides and prevent their dissolution into the electrolyte. In addition, it would be advantageous for the anchoring material to improve the electrical conductivity of the S electrode since it is relatively low.

Due to their unique and remarkable properties that meet the above-mentioned criteria, two-dimensional (2D) materials have been recognized to play an increasingly important role in developing efficient anchoring materials.^25−27^ Several 2D materials have been proposed as anchoring materials to suppress the shuttling effect, such as graphene, Ti_2_C MXene terminated with OH, F, and S,^28^ porous vanadium nitride nanoribbon with graphene,^29,30^ and borophosphorene.^31^ In this regard, two new members of hybrid 2D graphene-like nanosheets, B_2_C_4_P_2_ and B_3_C_2_P_3_, predicted recently based on density functional theory (DFT) calculations, are greatly interesting.^32^ These nanosheets have structural and thermodynamic stability and could be fabricated through carbon doping of boron phosphide. B_2_C_4_P_2_ is metallic, while B_3_C_2_P_2_ is a moderate band gap semiconductor (0.35 eV), suggesting good conductivity and high carrier mobility. It also has a planar lattice, a critical feature in constructing batteries with a fast charge/discharge rate, proposing its potential as an anchoring material. Therefore, comprehensive first-principles calculations are employed to reveal the promise of the nanosheets B_2_C_4_P_2_ and B_3_C_2_P_3_ as anchoring materials for Li–S batteries.

## Computational Methods

All the density functional theory (DFT) calculations are performed using the Vienna Ab initio Simulation Package.^33,34^ The plane-wave cutoff energy is set to 500 eV combined with an exchange–correlation functional of the generalized gradient approximation (Perdew–Burke–Ernzerhof flavor). The change in total energy and maximum atomic force converged to 10–^6^ and 0.01 eV Å^–1^, respectively. A 3 × 3 × 1 supercell and 5 × 5 × 1 Monkhorst–Pack grid of *k*-points were used for geometry relaxation. 6 × 6 × 1 and 9 × 9 × 1 *k*-meshes were employed for self-consistent and density of states (DOS) calculations, respectively. The DFT-D3 approach^35^ accurately accounts for the van der Waals forces. A vacuum of 18 Å eliminates spurious interlayer interactions due to periodicity in the out-of-plane direction. Bader charge analysis is used to quantify the charge transfer during the LiPS interaction with the sheets. The binding energy (*E*_b_) and charge density difference (ρ_b_) are determined using
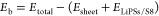
1
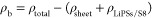
2where *E*_total_, *E*_sheet_, and *E*_LiPSs/S8_ correspond to the total energies of B_2_C_4_P_2_/B_3_C_2_P_3_ sheets with adsorbed LiPS/S_8_, pristine B_2_C_4_P_2_/B_3_C_2_P_3_ sheets, and isolated LiPS/S_8_ species, respectively. Similarly, ρ_total_, ρ_sheet_, and ρ_LiPSs/S8_ represent the charge densities of B_2_C_4_P_2_/B_3_C_2_P_3_ sheets with adsorbed LiPS/S_8_, pristine B_2_C_4_P_2_/B_3_C_2_P_3_ sheets, and isolated LiPS/S_8_ species, respectively.

## Results and Discussion

**Figure 1:**
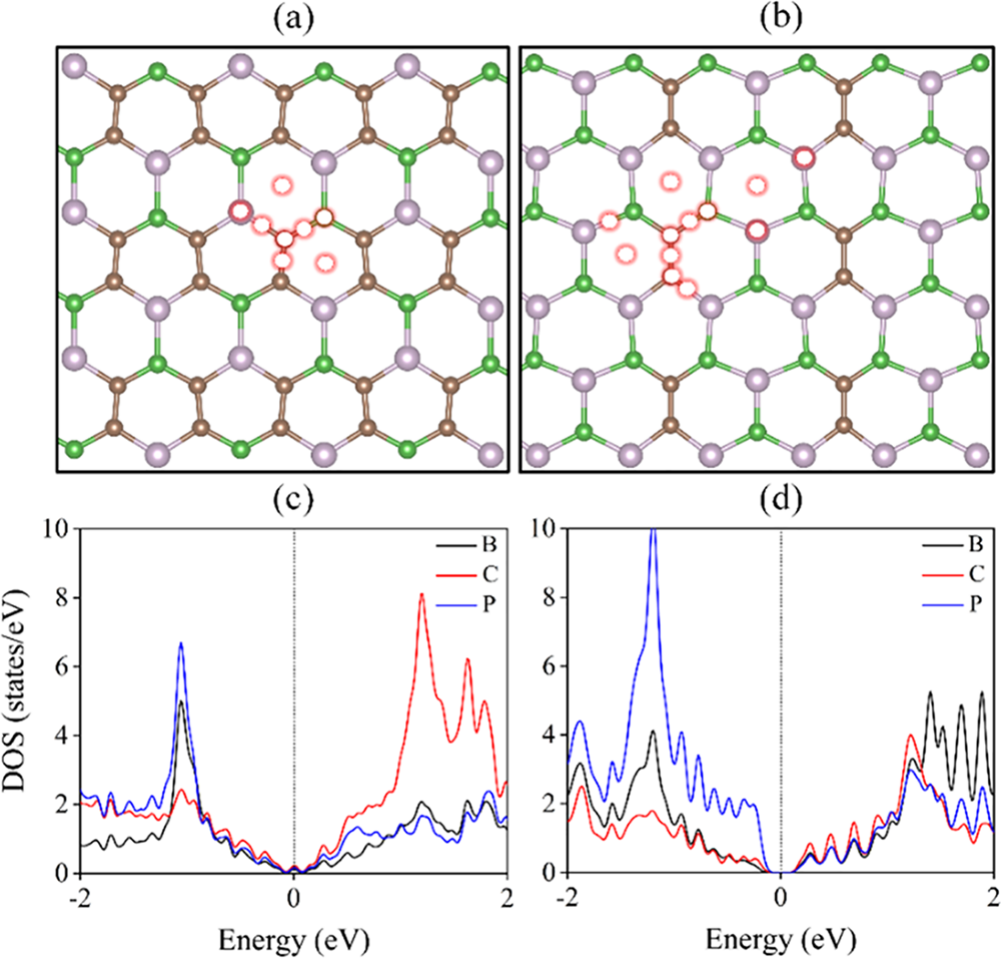
(a, b) Optimized 3 × 3 × 1 supercell of nanosheets B_2_C_4_P_2_ and B_3_C_2_P_3_. (c, d) Calculated DOS at the PBE level. The brown, green, and purple spheres represent carbon, boron, and phosphorous atoms, respectively. The red hollow circle defines the possible sites tested for the adsorption of Li_2_S*_x_*.

The optimized lattice parameters of the honeycomb nanosheets B_2_C_4_P_2_ and B_3_C_2_P_3_ are *a* = 5.63 Å and *b* = 4.94 Å for B_2_C_4_P_2_ and *a* = 6.06 Å and *b* = 5.20 Å for B_3_C_2_P_3_, in agreement with those reported in previous work.^32^ Both dynamic stability and thermal stability of B_2_C_4_P_2_ and B_3_C_2_P_3_ were confirmed before in the literature.^32^ The optimized 3 × 3 × 1 supercells of B_2_C_4_P_2_ and B_3_C_2_P_3_ are presented in Figure 1a,b. The partial density of states (DOS) of B_2_C_4_P_2_ (Figure 1c) demonstrates a strong hybridization between the states of phosphorus, boron, and carbon atoms, giving an aggregate contribution in the vicinity of the Fermi level. On the other hand, the partial DOS of B_3_C_2_P_3_ (Figure 1d) shows that phosphorus mainly contributes to the valence band maximum constitution, while the conduction band minimum is formed as a consequence of the hybridization of states from boron, carbon, and phosphorus atoms.

**Figure 2:**
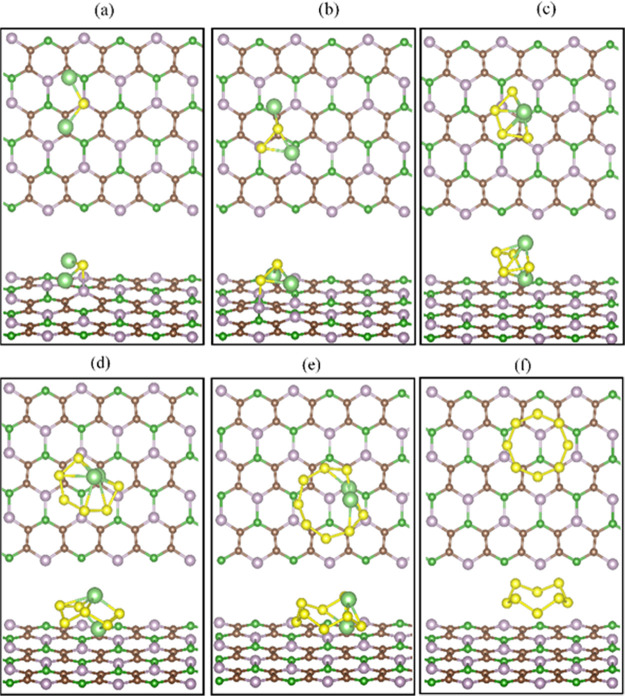
Optimized structures of (a) Li_2_S, (b) Li_2_S_2_, (c) Li_2_S_4_, (d) Li_2_S_6_, (e) Li_2_S_8_, and (f) S_8_ adsorbed on the nanosheet B_2_C_4_P_2_. The yellow, light green, brown, green, and purple solid spheres represent the sulfur, lithium, carbon, boron, and phosphorus atoms, respectively.

**Figure 3:**
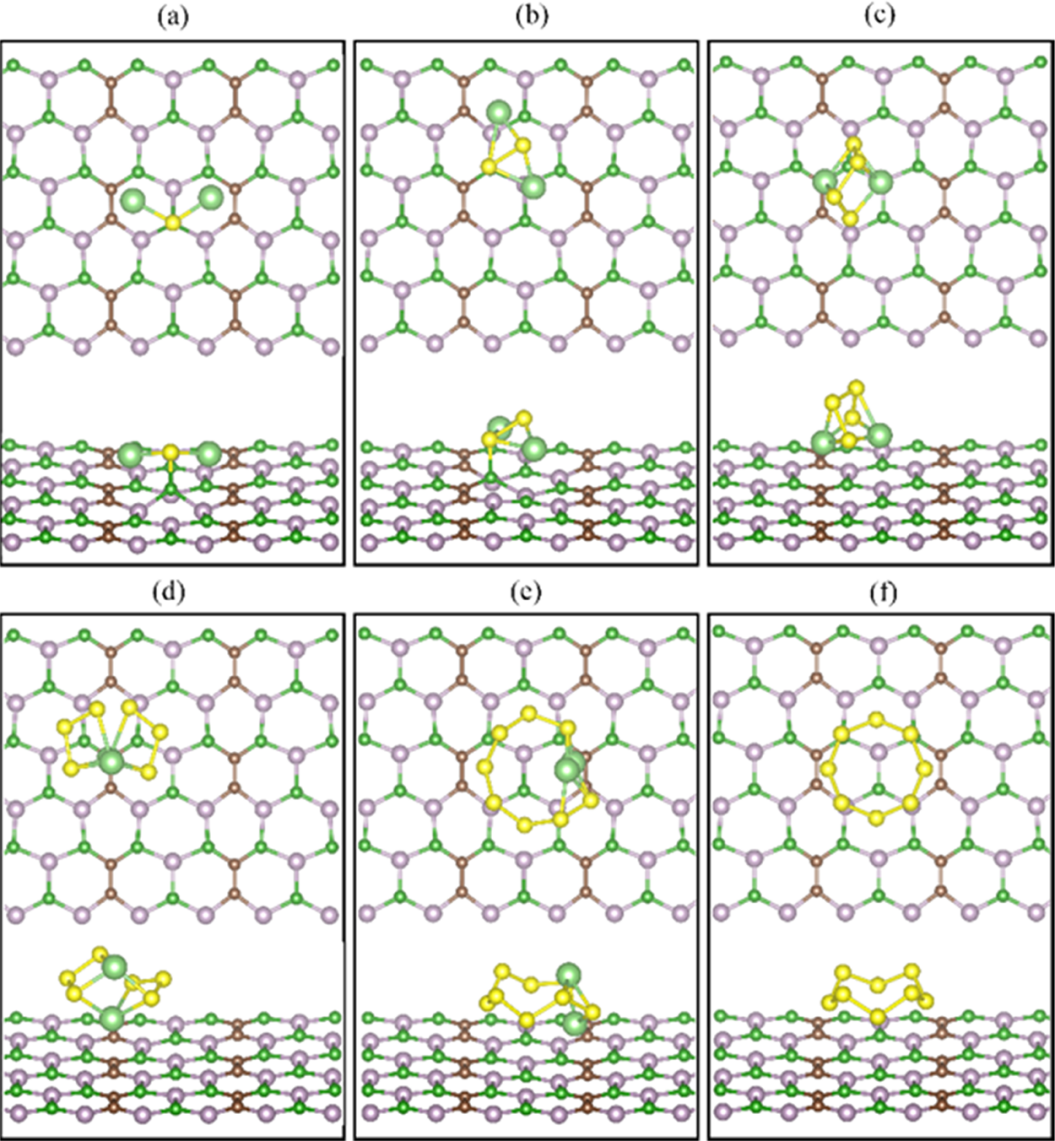
Optimized structures of (a) Li_2_S, (b) Li_2_S_2_, (c) Li_2_S_4_, (d) Li_2_S_6_, (e) Li_2_S_8_, and (f) S_8_ adsorbed on the nanosheet B_3_C_2_P_3_. The yellow, light green, brown, green, and purple solid spheres represent the sulfur, lithium, carbon, boron, and phosphorus atoms, respectively.

Polysulfides considerably impair the capacity and cycling stability of Li–S batteries. Through geometry optimization, we obtained the most stable structures of the Li_2_S*_x_* (*x* = 1, 2, 4, 6, 8) and S_8_ clusters formed at the S electrode. As the number of sulfur atoms increases, high-order LiPSs can be ionized and risk transportation back to the Li electrode in the recharging process, resulting in the shuttle effect.^2^ As evident from Figure 1a,b, the nanosheets possess multiple possible adsorption sites, as shown in Figure 1a,b. All rotational and translational configurations of LiPSs and S_8_ are considered on these sites, whereas the most stable configurations and the corresponding binding energies are presented in Figures 2 and 3 and Table 1, respectively. We have found that the nanosheet B_2_C_4_P_2_ and B_3_C_2_P_3_ strongly bind Li_2_S and Li_2_S_2_, whereas for higher ordered LiPSs and S_8_, the binding energies are lower but above 0.74 eV. The noticeable trend in the binding energy values is directly reflected in the distance between the polysulfides and the nanosheets. Li_2_S binds at 2.13 Å from B_2_C_4_P_2_ and 1.89 Å from B_3_C_2_P_3_, indicating chemical bonding. Such distances are shorter than those obtained for the other LiPSs and S_8_ that are physiosorbed; therefore, the binding energies are higher for Li_2_S. The calculated binding energies (see Table 1) are larger than those of graphene^20^ and comparable to those of blue phosphorene.^36^ Compared to graphene, the noticeable increase in binding energies for B_2_C_4_P_2_ and B_3_C_2_P_3_ can be understood by examining the bonding within the nanosheets. In the case of graphene, all the polysulfide physisorbed through dispersion van der Waals interactions, while in B_2_C_4_P_2_ and B_3_C_2_P_3_, the electronegativity difference between C, P, and B causes electrons in the C–P, C–B, and B–P bonds to be unequally shared, and binding through bond dipoles is also possible, which makes the van der Waals interactions stronger. Together, these findings suggest the better performance of the studied nanosheets to counter the shuttle effect.

**Table 1:** Binding Energies (eV) of LiPSs and S_8_ over B_2_C_4_P_2_, B_3_C_2_P_3_, Graphene, and Blue Phosphorene^a^

	B_2_C_4_P_2_	B_3_C_2_P_3_	graphene^20^	blue phosphorene^36^
Li_2_S	–2.22 (2.13)	–2.43 (1.89)	–0.50	–2.50
Li_2_S_2_	–1.31 (2.53)	–1.71 (2.31)	–0.50	–1.50
Li_2_S_4_	–0.76 (3.04)	–0.83 (2.90)	–0.60	–1.10
Li_2_S_6_	–0.93 (2.78)	–0.93 (2.81)	–0.70	–0.90
Li_2_S_8_	–1.03 (2.60)	–1.09 (2.64)	–0.90	–1.00
S_8_	–0.75 (3.2)	–0.74 (3.2)	–0.70	–0.50

a The separation distances (Å) of LiPSs and S_8_ over B_2_C_4_P_2_ and B_3_C_2_P_3_ are given in parenthesis.

Moreover, also, the S_8_ cluster binds with the surface with binding energies of 0.75 and 0.74 eV for B_2_C_4_P_2_ and B_3_C_2_P_3_, respectively. The interaction is weak due to the absence of Li atoms (in all LiPSs, the Li atoms interact with the surfaces). The S_8_ cluster orients parallel to the surface with a distance of 3.20 Å, which is also captured in the previous studies for similar 2D materials.^28^ An increase in binding energy with decreasing S content makes the discharge process over the surface possible. Also, we calculated the Li–S bond length of all LiPSs after adsorption on the nanosheets. In the case of B_2_C_4_P_2_, the average Li–S bond lengths increase compared to those of the free clusters, 0.24, 0.09, 0.13, 0.14, and 0.05 Å for Li_2_S, Li_2_S_2_, Li_2_S_4_, Li_2_S_6_, and Li_2_S_8_, respectively. Similarly, for the nanosheet B_3_C_2_P_3_, the changes in the Li–S bond lengths are 0.20, 0.21, 0.01, 0.14, and 0.04 Å, respectively. In the case of a higher S content, the change in bond lengths is smaller than that for the Li_2_S and Li_2_S_2_ clusters. An increased Li–S bond length reflects the weakening of the bond, governed by the strength of the cluster–surface interaction, and could help facilitate the discharge process.

**Figure 4:**
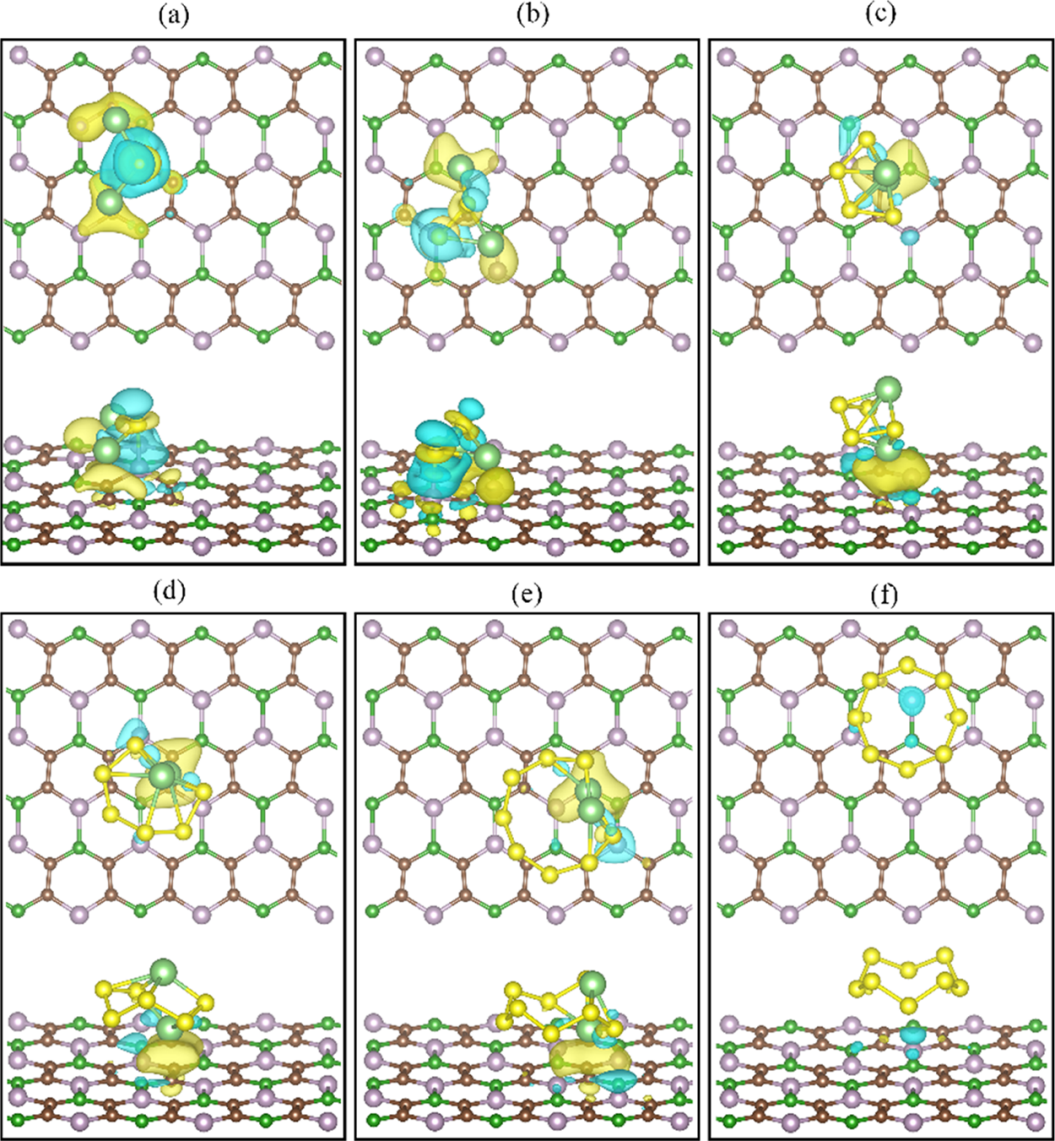
Charge density difference after (a) Li_2_S, (b) Li_2_S_2_, (c) Li_2_S_4_, (d) Li_2_S_6_, (e) Li_2_S_8_, and (f) S_8_ adsorption on the nanosheet B_2_C_4_P_2_. The cyan and yellow regions indicate charge depletion and accumulation, respectively (isosurface value 4 × 10^–3^ e Å^–3^).

**Figure 5:**
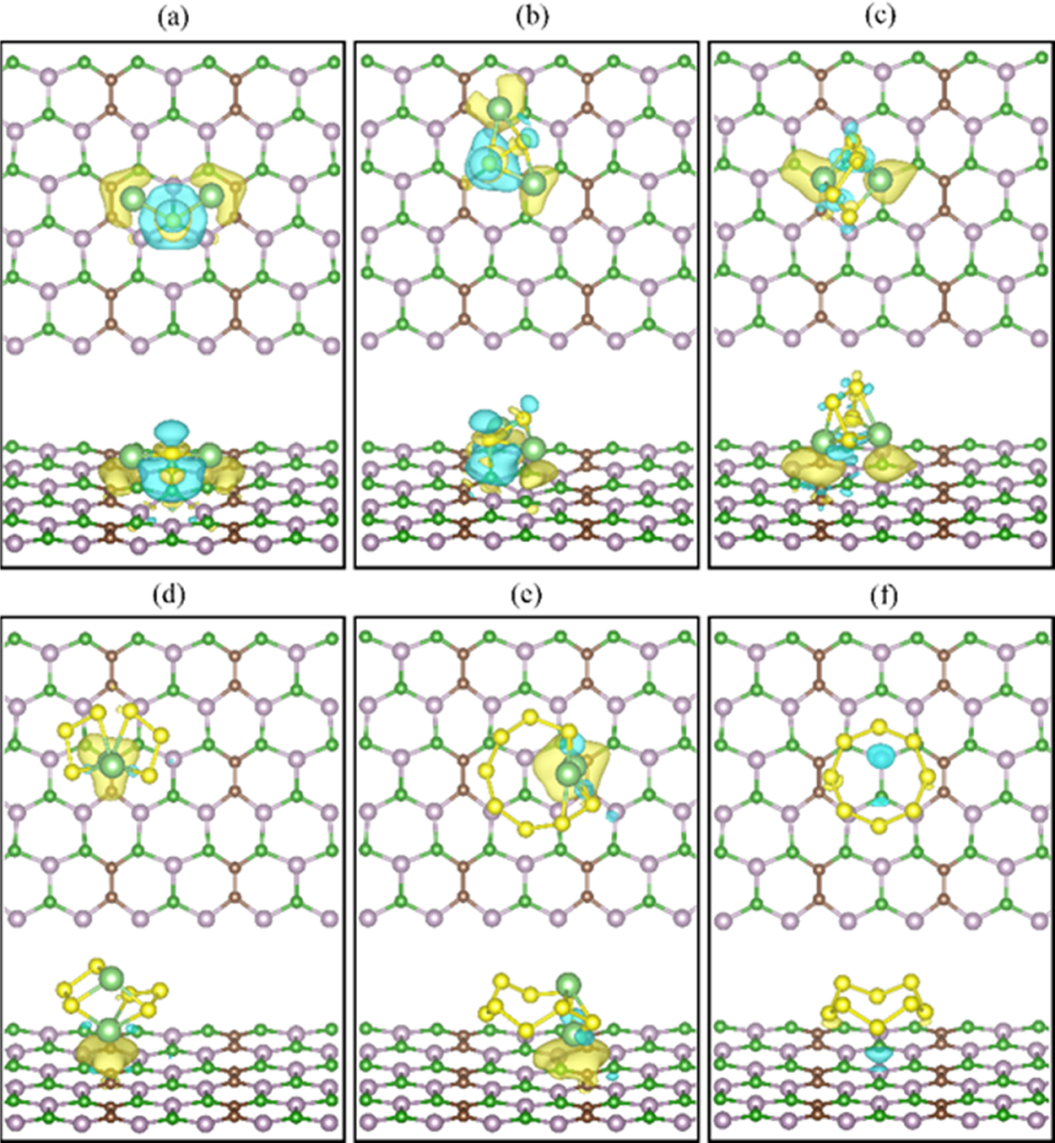
Charge density difference after (a) Li_2_S, (b) Li_2_S_2_, (c) Li_2_S_4_, (d) Li_2_S_6_, (e) Li_2_S_8_, and (f) S_8_ adsorption on the nanosheet B_3_C_2_P_3_. The cyan and yellow regions indicate charge depletion and accumulation, respectively (isosurface value 4 × 10^–3^ e Å^–3^).

We performed a Bader charge analysis to better understand the nanosheets’ binding mechanisms. The results show that the LiPSs donate charge to the nanosheets B_2_C_4_P_2_ and B_3_C_2_P_3_ (see Table 2). The charge is mainly accumulated (depleted) on S (Li) before adsorption and substantially transferred to the sheet after adsorption. The Bader charge values indicate that Li_2_S, Li_2_S_2_, and Li_2_S_4_ donate considerable charge to the surface, agreeing with their high binding strength and chemical bonding nature. The charge donation from the higher LiPSs is negligible. The charge distribution between the LiPSs and B_2_C_4_P_2_/B_3_C_2_P_3_ is calculated by the charge density differences, as shown in Figures 4 and 5. Electrons are mainly accumulated between Li and S atoms nearest to the nanosheets. Among the LiPSs, Li_2_S has the most apparent charge redistribution, consistent with its higher binding energies on both nanosheets. The two lithium atoms facing the surface cause the high charge transfer values and has the shortest interaction distances (2.13 and 1.89 Å for B_2_C_4_P_2_ and B_3_C_2_P_3_, respectively). A very similar charge transfer effect is found for Li_2_S_2_. On the contrary, Li_2_S_4_ (and the higher LiPSs) shows much lower charge transfer with longer interaction distances at 3.04 Å and 2.90 Å in the case of B_2_C_4_P_2_ and B_3_C_2_P_3_, respectively.

**Table 2:** Charge Transferred (in *e*) from LiPSs and S_8_ Clusters to the Nanosheets after Adsorption

	Li_2_S	Li_2_S_2_	Li_2_S_4_	Li_2_S_6_	Li_2_S_8_	S_8_
B_2_C_4_P_2_	–0.74	–0.60	–0.05	–0.04	–0.01	0.03
B_3_C_2_P_3_	–0.55	–0.45	–0.12	–0.02	–0.02	0.04

**Figure 6:**
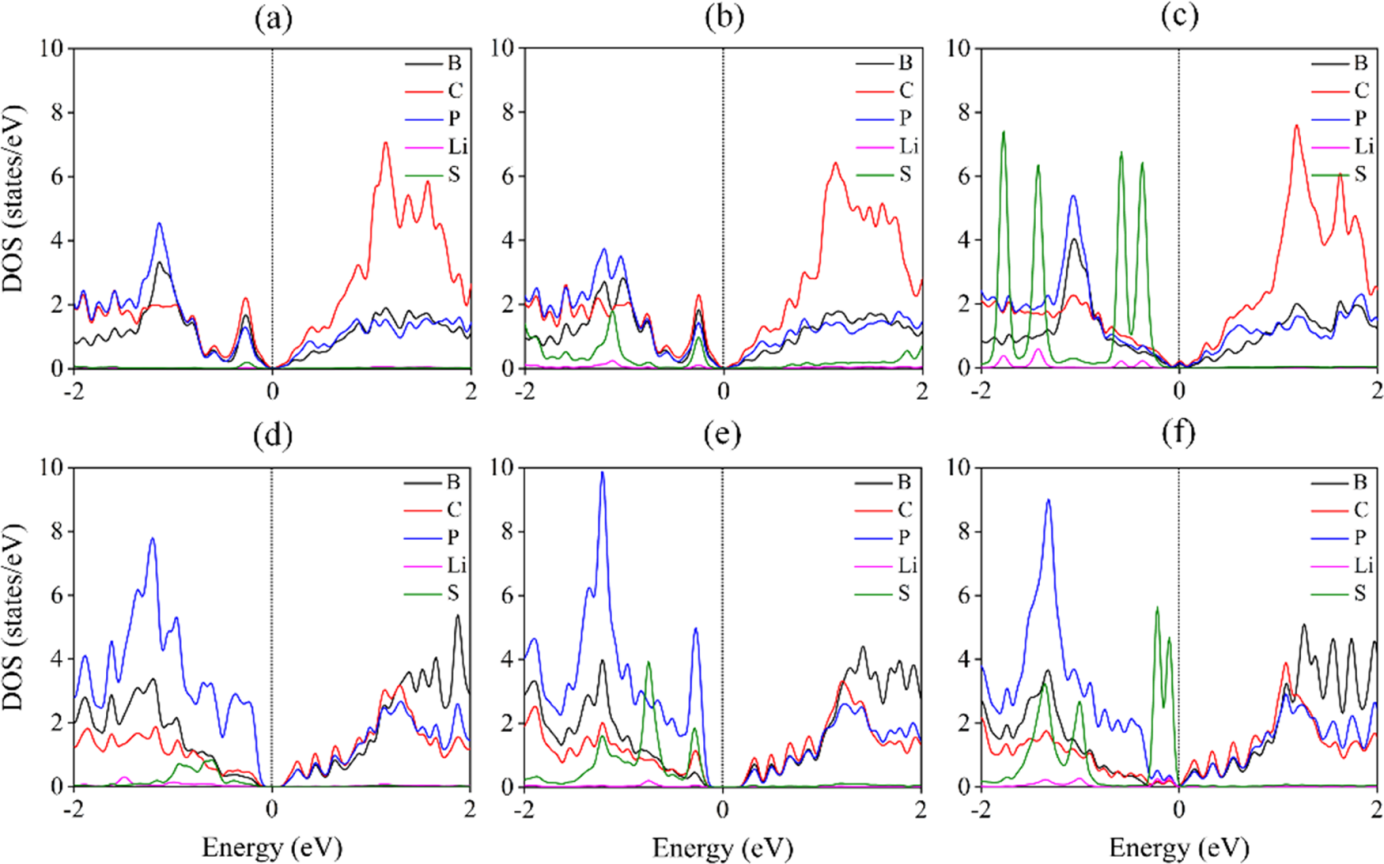
Calculated DOS of (a and d) Li_2_S, (b and e) Li_2_S_2_, and (c and f) Li_2_S_4_ adsorbed on B_2_C_4_P_2_ (1st row) and B_3_C_2_P_3_ (2nd row), respectively.

Since the conductive behavior of the anchoring material is an additional beneficial parameter for the performance of the S electrode in Li–S batteries, the density of states after adsorption of Li_2_S, Li_2_S_2_, and Li_2_S_4_ on the nanosheets is presented in Figure 6. After adsorption, the B_2_C_4_P_2_ nanosheet preserves its metallic properties, whereas the electronic band gap of B_3_C_2_P_3_ reduces to 0.15 with Li_2_S adsorption, 0.20 eV in the case of Li_2_S_2_, and 0 eV in the case of Li_2_S_4_ due to the donation of electrons to the nanosheet. The band gap directly influences the electrical conductivity (σ) according to the expression σ = exp(−Δ*E*/*k*_B_*T*),^37^ where Δ*E* is the band gap. Therefore, the small band gap would significantly improve the conductivity of the sulfur anode to enhance the performance of Li–S batteries. The partial density of states for Li_2_S-, Li_2_S_2_-, and Li_2_S_4_-adsorbed systems is shown in Figure 6. The contribution of the S and Li atoms in the partial density of states around the Fermi level is due to the charge transfer between the clusters and the nanosheets (Figure 6).

**Figure 7:**
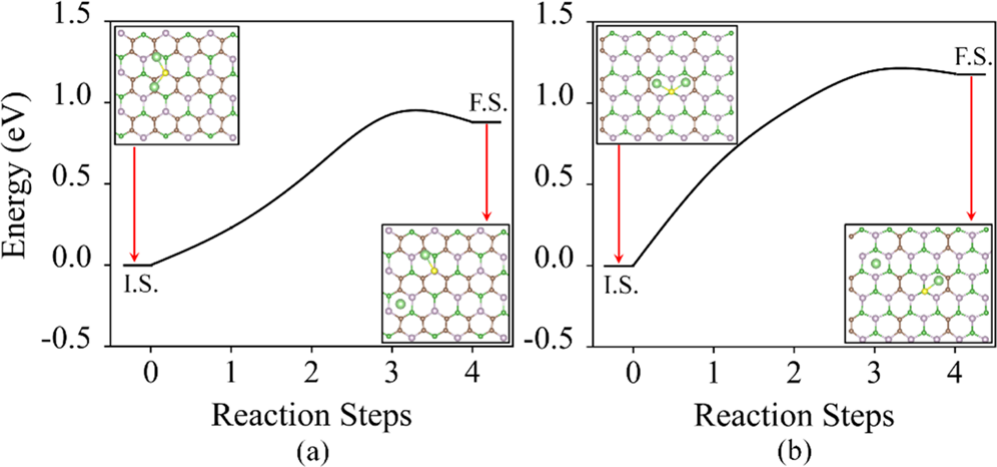
Energy barrier for decomposition of Li_2_S on (a) B_2_C_4_P_2_ and (b) B_3_C_2_P_3_. The insets show the structure of the initial state (IS) and final state (FS). The black lines connecting the IS and FS are only to guide the eye.

During the recharging phase of Li–S batteries, the low-order sulfur cluster Li_2_S begins to disintegrate and oxidize to high-order sulfur clusters, eventually forming S_8_ clusters. Moreover, the catalysis of the disintegration of Li_2_S clusters on the substrate surface is essential for achieving high capacity and Columbic efficiency.^38^ We have calculated the Li_2_S → LiS + Li^+^ + e^–^ barrier energy through the climbing image nudged elastic band method. The barrier energy can explain the energy required to break the bond (Li–S) on the surface. The initial and final states for B_2_C_4_P_2_ and B_3_C_2_P_3_ are shown in the inset of Figure 7a,b. The energy required for the reaction is 0.93 and 1.20 eV over B_2_C_4_P_2_ and B_3_C_2_P_3_, respectively. This barrier energy value of B_2_C_4_P_2_ and B_3_C_2_P_3_ is similar to that of other 2D materials.^32,34^

## Conclusions

In summary, the promise of 2D nanosheets B_2_C_4_P_2_ and P_3_C_2_P_3_ as anchoring materials has been systematically explored using DFT calculations. Both nanosheets show significant adsorption energies of LiPSs due to the charge transfer from LiPSs to the nanosheets, favorable to counter the shuttle effect in Li–S batteries. B_2_C_4_P_2_ possesses a metallic character, whereas B_3_C_2_P_3,_ upon LiPS adsorption, undergoes band gap reduction, which enhances the electrical conductivity of the sulfur electrode as necessary in Li–S batteries. Moreover, the calculated barrier energy of Li^+^ detachment is found to be 0.92 and 1.20 eV for B_2_C_4_P_2_ and B_3_C_2_P_3_, respectively, indicating fast diffusion as compared to blue phosphorene and metal sulfides. These findings would encourage developing and synthesizing the nanosheets B_2_C_4_P_2_ and B_3_C_2_P_3_ as high-performance anchoring materials.
